# 2,6-Dimethyl-4-(1,3,4-oxadiazol-2-yl)quinoline

**DOI:** 10.1107/S1600536810048683

**Published:** 2010-11-27

**Authors:** Artyom G. Kashaev, Anatoliy V. Zimichev, Victor B. Rybakov, Yurij N. Klimochkin, Margarita N. Zemtsova

**Affiliations:** a Samara State Technical University, Molodogvardeyskay Str. 244, 443100 Samara, Russian Federation; b Department of Chemistry, Moscow State University, 119992 Moscow, Russian Federation

## Abstract

The title compound, C_13_H_11_N_3_O, a potential chemotherapeutic agent, contains a essential planar [maximum deviation = 0.0144 (14) Å] quinoline moiety. The quinoline ring system and the five-membered heterocycle form a dihedral angle of 7.81 (6)°. In the crystal, inter­molecular non-classical C—H⋯N hydrogen bonding is present.

## Related literature

For general background to the use of compounds containing a quinoline fragment as chemotherapeutical agents, see: Kaila *et al.* (2007 ▶); Vaitilingam *et al.* (2004 ▶). 
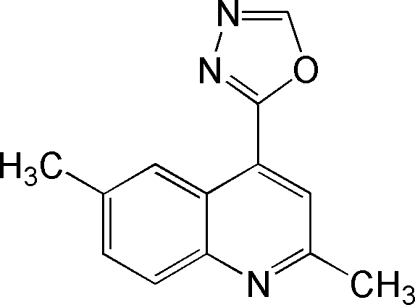


## Experimental

### 

#### Crystal data

C_13_H_11_N_3_O
*M* *_r_* = 225.25
Monoclinic, 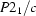

*a* = 15.5372 (14) Å
*b* = 9.7546 (7) Å
*c* = 7.3984 (5) Å
β = 100.64 (1)°
*V* = 1102.02 (15) Å^3^
*Z* = 4
Cu *K*α radiation
μ = 0.73 mm^−1^
*T* = 295 K
0.20 × 0.20 × 0.20 mm

#### Data collection

Enraf–Nonius CAD-4 diffractometer
Absorption correction: refined from Δ*F* (Walker & Stuart, 1983 ▶) *T*_min_ = 0.391, *T*_max_ = 0.865
2236 measured reflections
2236 independent reflections
1855 reflections with *I* > 2σ(*I*)
1 standard reflections every 60 min  intensity decay: 1%

#### Refinement

*R*[*F* ^2^ > 2σ(*F* ^2^)] = 0.042
*wR*(*F* ^2^) = 0.130
*S* = 1.07
2236 reflections
156 parameters
H-atom parameters constrained
Δρ_max_ = 0.18 e Å^−3^
Δρ_min_ = −0.14 e Å^−3^

### 

Data collection: *CAD-4 EXPRESS* (Enraf–Nonius, 1994 ▶); cell refinement: *CAD-4 EXPRESS*; data reduction: *XCAD4* (Harms & Wocadlo, 1995 ▶); program(s) used to solve structure: *SHELXS97* (Sheldrick, 2008 ▶); program(s) used to refine structure: *SHELXL97* (Sheldrick, 2008 ▶); molecular graphics: *ORTEP-3* (Farrugia, 1997 ▶); software used to prepare material for publication: *WinGX* (Farrugia, 1999 ▶).

## Supplementary Material

Crystal structure: contains datablocks global, I. DOI: 10.1107/S1600536810048683/ds2065sup1.cif

Structure factors: contains datablocks I. DOI: 10.1107/S1600536810048683/ds2065Isup2.hkl

Additional supplementary materials:  crystallographic information; 3D view; checkCIF report

## Figures and Tables

**Table 1:** Hydrogen-bond geometry (Å, °)

*D*—H⋯*A*	*D*—H	H⋯*A*	*D*⋯*A*	*D*—H⋯*A*
C13—H13⋯N15^i^	0.93	2.59	3.523 (2)	178

Symmetry code: (i) 
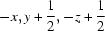
.

**Fig. 1.**
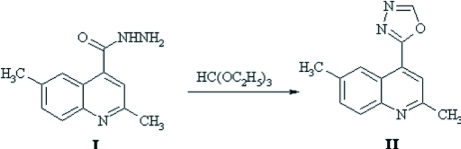
Synthesis of the title compound.

**Fig. 2.**
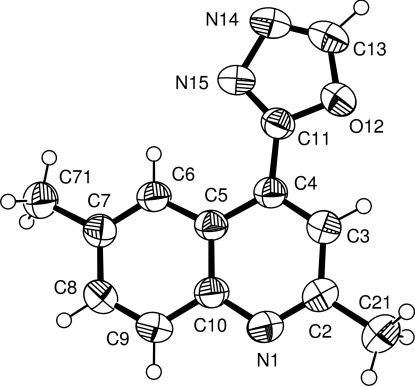
ORTEP-3 (Farrugia, 1997) plot of molecular structure of the title compound showing the atom-numbering scheme. Thermal displacement ellipsoids are drawn at the 50% probability level. H atoms are presented as a small spheres of arbitrary radius.

## supplementary crystallographic information

### Comment

The compounds containing a fragment of quinoline ring, are widely used as chemotherapeutical agents (Vaitilingam *et al.*, 2004; Kaila *et al.*, 2007). Synthesis of new quinoline derivatives and study of its properties to be of interest in theoretic and practical aspects as well. The 2,6-dimethyl-4-(1,3,4-oxadiazol-2-yl)quinoline II was synthesized from triethyl orthoformate and hydrazide 2,6-dimethyl-4-quinoline carboxylic acid I (Fig. 1).

In the crystal structure is found non-classical intermolecular hydrogen bond - C13–H13···N15^i^, where contacts H13···N15^i^ = 2.594 Å, C13···N15^i^ = 3.523 (2)Å and angle C13–H13···N15^i^ = 178°. Symmetry code:(i) -*x*, *y* + 1/2, -*z* + 1/2. The short intramolecular contacts C6–H6···N15 (H6···N15 = 2.370 Å) and C3–H3···O12 (H3···O12 = 2.372 Å) are obliged by conjugation of oxadiazol and guinoline moieties - in title molecule, guinoline moiety is planar (max deviation of C7 = 0.0144 (14) Å) and essential planar oxadiazol moiety form dihedral angle 7.81 (6)° (Fig. 2).

### Experimental

A solution of triethyl orthoformate (60 mmol) and 2,6-dimethyl-4-(1,3,4-oxadiazol-2-yl)quinoline (5 mmol) was refluxed for 20 h. Ester was removed in a vacuum. Recrystallization of the crude product from ethanol gave 0.89 g of colourless crystals. Yield 79%, mp 448-449 K.

IR, ν, cm^-1^: 3116 (C–H, oxadiazol), 1751 (CO), 1600 (C═C), 1508 (C═ N). MS, m/*z*: 225 (100) [*M*]^+^, 210 (17), 184 (11), 156 (32), 115 (15), 89 (8), 63 (9). ^1^H NMR, δ: 2.51 s (3*H*, 6-CH_3_), 2.71 s (3*H*, 2-CH_3_), 7.63 d (1*H*, J = 8.80, 7-H), 7.89 s (1*H*, 3-H), 7.92 d (1*H*, J = 8.80, 8-H), 8.71 s (1*H*, 5-H), 9.52 s (1*H*, C–H oxadiazol). Anal. calc. for C_13_H_11_N_3_O, %: C 69.32; H 4.92; N 18.66. Found, %: C 69.27; H 4.83; N 18.61.

Single crystals for *X*-ray analysis were obtained by slow evaporation of an ethanol. IR spectrum was recorded (in KBr) on Shimadzu FTIR-8400S. Mass spectrum was measured on Finnigan Trance DSQ spectrometer. ^1^H NMR spectrum was obtained in *DMSO*-d_6_ on Bruker AM 300 (300 MHz), using *TMS* as internal standard. Elemental composition was determined on Euro Vector EA-3000 elemental analyzer.

### Refinement

C-bound H-atoms were placed in calculated positions (C–H 0.93 Å & 0.97 Å) and refined as riding, with *U*_iso_(H) = 1.2*U*_eq_(C).

### Crystal data

**Table:**

C_13_H_11_N_3_O	*F*(000) = 472
*M**_r_* = 225.25	*D*_x_ = 1.358 Mg m^−^^3^
Monoclinic, *P*2_1_/*c*	Melting point = 448–449 K
Hall symbol: -P 2ybc	Cu *K*α radiation, λ = 1.54184 Å
*a* = 15.5372 (14) Å	Cell parameters from 25 reflections
*b* = 9.7546 (7) Å	θ = 36.3–39.9°
*c* = 7.3984 (5) Å	µ = 0.73 mm^−^^1^
β = 100.64 (1)°	*T* = 295 K
*V* = 1102.02 (15) Å^3^	Prism, colourless
*Z* = 4	0.20 × 0.20 × 0.20 mm

### Data collection

**Table:**

Enraf–Nonius CAD-4 diffractometer	1855 reflections with *I* > 2σ(*I*)
Radiation source: Fine-focus sealed tube	*R*_int_ = 0.0000
Graphite	θ_max_ = 74.9°, θ_min_ = 2.9°
Non–profiled ω scans	*h* = 0→19
Absorption correction: part of the refinement model (Δ*F*) (Walker & Stuart, 1983)	*k* = 0→12
*T*_min_ = 0.391, *T*_max_ = 0.865	*l* = −8→9
2236 measured reflections	1 standard reflections every 60 min
2236 independent reflections	intensity decay: 1%

### Refinement

**Table:**

Refinement on *F*^2^	Primary atom site location: structure-invariant direct methods
Least-squares matrix: full	Secondary atom site location: difference Fourier map
*R*[*F*^2^ > 2σ(*F*^2^)] = 0.042	Hydrogen site location: inferred from neighbouring sites
*wR*(*F*^2^) = 0.130	H-atom parameters constrained
*S* = 1.07	*w* = 1/[σ^2^(*F*_o_^2^) + (0.0669*P*)^2^ + 0.1662*P*] where *P* = (*F*_o_^2^ + 2*F*_c_^2^)/3
2236 reflections	(Δ/σ)_max_ = 0.005
156 parameters	Δρ_max_ = 0.18 e Å^−^^3^
0 restraints	Δρ_min_ = −0.14 e Å^−^^3^

### Special details

**Table:**

Geometry. All s.u.'s (except the s.u. in the dihedral angle between two l.s. planes) are estimated using the full covariance matrix. The cell s.u.'s are taken into account individually in the estimation of s.u.'s in distances, angles and torsion angles; correlations between s.u.'s in cell parameters are only used when they are defined by crystal symmetry. An approximate (isotropic) treatment of cell s.u.'s is used for estimating s.u.'s involving l.s. planes.
Refinement. Refinement of F^2^ against ALL reflections. The weighted R-factor wR and goodness of fit S are based on F^2^, conventional R-factors R are based on F, with F set to zero for negative F^2^. The threshold expression of F^2^ > 2σ(F^2^) is used only for calculating R-factors(gt) etc. and is not relevant to the choice of reflections for refinement. R-factors based on F^2^ are statistically about twice as large as those based on F, and R-factors based on ALL data will be even larger.

### Fractional atomic coordinates and isotropic or equivalent isotropic displacement parameters (Å^2^)

**Table:**

	*x*	*y*	*z*	*U*_iso_*/*U*_eq_	
N1	0.39891 (8)	0.63577 (13)	0.05252 (18)	0.0557 (3)	
C2	0.36926 (10)	0.75870 (16)	0.0823 (2)	0.0549 (4)	
C21	0.42870 (12)	0.87852 (19)	0.0735 (3)	0.0720 (5)	
H21A	0.4875	0.8464	0.0781	0.108*	
H21B	0.4271	0.9383	0.1758	0.108*	
H21C	0.4096	0.9276	−0.0393	0.108*	
C3	0.28466 (10)	0.77894 (16)	0.1206 (2)	0.0537 (4)	
H3	0.2663	0.8673	0.1417	0.064*	
C4	0.22912 (9)	0.67122 (14)	0.12717 (19)	0.0479 (3)	
C5	0.25819 (9)	0.53629 (15)	0.09414 (18)	0.0468 (3)	
C6	0.20838 (9)	0.41454 (15)	0.09503 (19)	0.0505 (3)	
H6	0.1513	0.4206	0.1158	0.061*	
C7	0.24197 (10)	0.28864 (15)	0.0662 (2)	0.0532 (4)	
C71	0.19028 (12)	0.15983 (16)	0.0748 (3)	0.0656 (4)	
H71A	0.1315	0.1829	0.0868	0.098*	
H71B	0.2171	0.1062	0.1789	0.098*	
H71C	0.1891	0.1078	−0.0358	0.098*	
C8	0.32817 (11)	0.28112 (16)	0.0302 (2)	0.0599 (4)	
H8	0.3515	0.1959	0.0097	0.072*	
C9	0.37777 (10)	0.39518 (17)	0.0249 (2)	0.0587 (4)	
H9	0.4340	0.3871	−0.0003	0.070*	
C10	0.34470 (9)	0.52589 (15)	0.0574 (2)	0.0508 (3)	
C11	0.14273 (9)	0.69987 (15)	0.1721 (2)	0.0500 (3)	
O12	0.11965 (7)	0.83379 (11)	0.18110 (16)	0.0631 (3)	
C13	0.03932 (11)	0.8249 (2)	0.2255 (3)	0.0688 (5)	
H13	0.0057	0.9013	0.2419	0.083*	
N14	0.01403 (9)	0.70333 (17)	0.2429 (2)	0.0739 (4)	
N15	0.08253 (9)	0.61910 (15)	0.2072 (2)	0.0651 (4)	

### Atomic displacement parameters (Å^2^)

**Table:**

	*U*^11^	*U*^22^	*U*^33^	*U*^12^	*U*^13^	*U*^23^
N1	0.0469 (6)	0.0603 (8)	0.0626 (8)	−0.0012 (5)	0.0168 (5)	0.0026 (6)
C2	0.0506 (8)	0.0585 (9)	0.0568 (8)	−0.0052 (6)	0.0135 (6)	0.0015 (6)
C21	0.0661 (10)	0.0651 (10)	0.0887 (13)	−0.0142 (8)	0.0241 (9)	0.0008 (9)
C3	0.0542 (8)	0.0518 (8)	0.0570 (9)	0.0007 (6)	0.0150 (6)	−0.0010 (6)
C4	0.0464 (7)	0.0516 (8)	0.0468 (8)	0.0019 (6)	0.0117 (6)	0.0008 (6)
C5	0.0456 (7)	0.0520 (8)	0.0442 (7)	0.0029 (6)	0.0119 (5)	0.0018 (5)
C6	0.0472 (7)	0.0558 (8)	0.0508 (8)	0.0007 (6)	0.0150 (6)	0.0008 (6)
C7	0.0561 (8)	0.0517 (8)	0.0532 (8)	0.0012 (6)	0.0134 (6)	0.0002 (6)
C71	0.0701 (10)	0.0550 (9)	0.0745 (11)	−0.0045 (8)	0.0207 (8)	−0.0047 (7)
C8	0.0602 (9)	0.0530 (8)	0.0695 (10)	0.0098 (7)	0.0195 (7)	0.0008 (7)
C9	0.0491 (8)	0.0616 (9)	0.0693 (10)	0.0082 (7)	0.0209 (7)	0.0009 (7)
C10	0.0463 (7)	0.0557 (8)	0.0522 (8)	0.0019 (6)	0.0142 (6)	0.0033 (6)
C11	0.0501 (7)	0.0510 (8)	0.0506 (8)	0.0051 (6)	0.0138 (6)	−0.0015 (6)
O12	0.0570 (6)	0.0550 (6)	0.0804 (8)	0.0078 (5)	0.0207 (5)	−0.0039 (5)
C13	0.0575 (9)	0.0735 (11)	0.0795 (12)	0.0173 (8)	0.0235 (8)	−0.0056 (9)
N14	0.0573 (8)	0.0778 (10)	0.0940 (11)	0.0120 (7)	0.0337 (7)	0.0009 (8)
N15	0.0538 (7)	0.0647 (8)	0.0840 (10)	0.0053 (6)	0.0315 (7)	0.0014 (7)

### Geometric parameters (Å, °)

**Table:**

N1—C2	1.3177 (19)	C7—C8	1.415 (2)
N1—C10	1.3677 (18)	C7—C71	1.499 (2)
C2—C3	1.408 (2)	C71—H71A	0.9600
C2—C21	1.499 (2)	C71—H71B	0.9600
C21—H21A	0.9600	C71—H71C	0.9600
C21—H21B	0.9600	C8—C9	1.358 (2)
C21—H21C	0.9600	C8—H8	0.9300
C3—C4	1.366 (2)	C9—C10	1.412 (2)
C3—H3	0.9300	C9—H9	0.9300
C4—C5	1.4271 (19)	C11—N15	1.286 (2)
C4—C11	1.4680 (19)	C11—O12	1.3596 (17)
C5—C6	1.4182 (19)	O12—C13	1.3508 (19)
C5—C10	1.4233 (19)	C13—N14	1.263 (2)
C6—C7	1.366 (2)	C13—H13	0.9300
C6—H6	0.9300	N14—N15	1.4075 (18)
			
C2—N1—C10	118.21 (12)	C7—C71—H71A	109.5
N1—C2—C3	121.95 (14)	C7—C71—H71B	109.5
N1—C2—C21	117.71 (14)	H71A—C71—H71B	109.5
C3—C2—C21	120.33 (14)	C7—C71—H71C	109.5
C2—C21—H21A	109.5	H71A—C71—H71C	109.5
C2—C21—H21B	109.5	H71B—C71—H71C	109.5
H21A—C21—H21B	109.5	C9—C8—C7	121.66 (14)
C2—C21—H21C	109.5	C9—C8—H8	119.2
H21A—C21—H21C	109.5	C7—C8—H8	119.2
H21B—C21—H21C	109.5	C8—C9—C10	120.58 (13)
C4—C3—C2	121.23 (14)	C8—C9—H9	119.7
C4—C3—H3	119.4	C10—C9—H9	119.7
C2—C3—H3	119.4	N1—C10—C9	117.26 (13)
C3—C4—C5	118.75 (13)	N1—C10—C5	123.86 (13)
C3—C4—C11	118.15 (13)	C9—C10—C5	118.88 (13)
C5—C4—C11	123.09 (12)	N15—C11—O12	111.76 (13)
C6—C5—C10	118.46 (13)	N15—C11—C4	131.21 (14)
C6—C5—C4	125.54 (12)	O12—C11—C4	117.03 (12)
C10—C5—C4	116.00 (13)	C13—O12—C11	102.38 (12)
C7—C6—C5	121.84 (13)	N14—C13—O12	113.79 (14)
C7—C6—H6	119.1	N14—C13—H13	123.1
C5—C6—H6	119.1	O12—C13—H13	123.1
C6—C7—C8	118.56 (14)	C13—N14—N15	105.60 (13)
C6—C7—C71	121.59 (13)	C11—N15—N14	106.47 (14)
C8—C7—C71	119.84 (14)		
			
C10—N1—C2—C3	0.7 (2)	C2—N1—C10—C5	−0.4 (2)
C10—N1—C2—C21	−178.99 (14)	C8—C9—C10—N1	179.04 (14)
N1—C2—C3—C4	−0.4 (2)	C8—C9—C10—C5	−0.6 (2)
C21—C2—C3—C4	179.24 (15)	C6—C5—C10—N1	179.88 (13)
C2—C3—C4—C5	−0.1 (2)	C4—C5—C10—N1	−0.1 (2)
C2—C3—C4—C11	178.58 (13)	C6—C5—C10—C9	−0.5 (2)
C3—C4—C5—C6	−179.61 (13)	C4—C5—C10—C9	179.53 (13)
C11—C4—C5—C6	1.8 (2)	C3—C4—C11—N15	−171.28 (16)
C3—C4—C5—C10	0.3 (2)	C5—C4—C11—N15	7.4 (3)
C11—C4—C5—C10	−178.28 (13)	C3—C4—C11—O12	8.1 (2)
C10—C5—C6—C7	1.6 (2)	C5—C4—C11—O12	−173.22 (12)
C4—C5—C6—C7	−178.43 (14)	N15—C11—O12—C13	0.07 (17)
C5—C6—C7—C8	−1.6 (2)	C4—C11—O12—C13	−179.46 (13)
C5—C6—C7—C71	177.53 (14)	C11—O12—C13—N14	0.0 (2)
C6—C7—C8—C9	0.4 (2)	O12—C13—N14—N15	−0.1 (2)
C71—C7—C8—C9	−178.70 (15)	O12—C11—N15—N14	−0.13 (18)
C7—C8—C9—C10	0.7 (3)	C4—C11—N15—N14	179.32 (15)
C2—N1—C10—C9	179.94 (13)	C13—N14—N15—C11	0.14 (19)

### Hydrogen-bond geometry (Å, °)

**Table:**

*D*—H···*A*	*D*—H	H···*A*	*D*···*A*	*D*—H···*A*
C13—H13···N15^i^	0.93	2.59	3.523 (2)	178

Symmetry codes: (i) −*x*, *y*+1/2, −*z*+1/2.
